# An elusive case of digital ischemia in a patient with Rheumatoid Arthritis

**DOI:** 10.12669/pjms.344.15281

**Published:** 2018

**Authors:** Waleed Azam, Uzma Erum, Asif Jan Muhammad

**Affiliations:** 1 Dr. Waleed Azam, MBBS, FCPS Trainee. Medicine Department, Patel Hospital, ST-18, Block-4, Gulshan-e-Iqbal, Karachi, Pakistan; 2 Dr. Uzma Erum, MBBS, FCPS (Medicine). Medicine Department, Patel Hospital, ST-18, Block-4, Gulshan-e-Iqbal, Karachi, Pakistan; 3 Dr. Asif Jan Muhammad, MBBS, MCPS, MRCP (UK). Medicine Department, Patel Hospital, ST-18, Block-4, Gulshan-e-Iqbal, Karachi, Pakistan

**Keywords:** Erythromelalgia, Essential thrombocytosis, Raynaud’s phenomenon, Rheumatoid arthritis

## Abstract

Essential thrombocytosis (ET) has rarely been reported with autoimmune rheumatic disorders. We report a case of young female, diagnosed case of Rheumatoid arthritis (RA), who had been overlooked for her raised platelet counts. Later her symptoms of impending digital gangrene led to an active search for her thrombocytosis. JAK2 mutation came out to be positive and she was diagnosed as ET associated with RA. She was treated with Hydroxyurea and Aspirin, in addition to her RA treatment. Patient responded well to the treatment and her platelet counts have been gradually improved, however, she developed gangrene of toe, for which amputation of distal phalanx of toe and nail excision was done, later in the disease course.

## INTRODUCTION

There is increasing evidencethat abnormal expression of pro-inflammatory cytokines in chronic inflammation may lead to unregulated clonal proliferation.^1^ Although, thrombocytosis may occur in Rheumatoid arthritis (RA) as reflection of high inflammatory state, true primary or essential thrombocytosis (ET) is extremely rare. An estimated prevalence of 30 per 100000 people has been reported for ET in general population.^2^ However, no causal relationship is known to exist between ET and RA. We report an unusual case of essential thrombocythemia that manifested as digital ischemia in a patient already diagnosed as RA. This leads to an intriguing possibility to speculate a substantial link between malignancy and autoimmune inflammatory disorders.

## CASE REPORT

**Fig.1:**
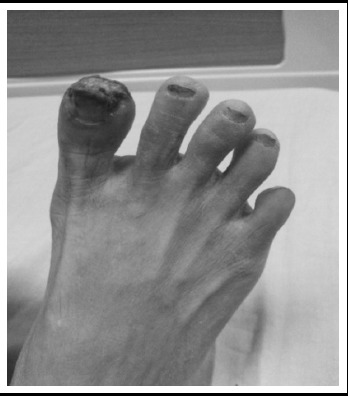
Digital ulceration and Gangrene of toes

**Fig.2:**
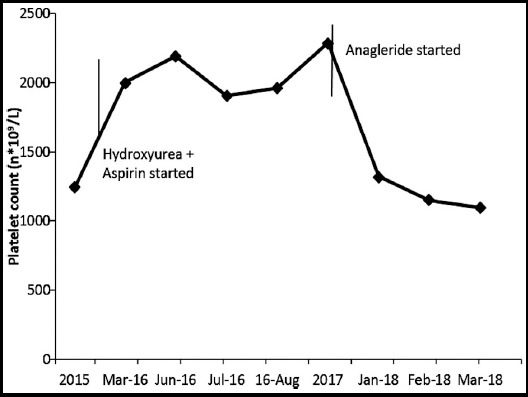
Platelet pattern.

We report a case of 38 year old lady, diagnosed case of Rheumatoid arthritis (RA) for two years. Apart from the symptoms of joint pains, there was history of Raynaud’s phenomenon as well. There were no systemic complaints and she remained well on treatment for RA, i.e. Hydroxychloroquin, Leflonamide and NSAID as per need. She presented to us, for the first time in June 2016, with complaints of blackish discoloration of right big toe and left 4^th^ toe for 1.5 years. She initially developed a small ulcerated lesion over one of her toes 1.5 year back, that was attributed to RA associated vasculitis and her treatment was modified in accordance with the suspected diagnosis, which included Aspirin, Nefidipine, and Prednisolone. There was no complaint of pain, itching or temperature change at that time and she remained static until a week prior to her presentation to us. This time she had moderate intensity pain in her toes, specifically right big toe and left 4^th^ toe which turned blackish in color. (Fig.1) She was admitted in ward on this occasion. Examination showed a young lady, BMI 21 Kg/m^2^, Pulse- 90bpm, BP - 150/80 mmHg, temperature – 99°F, RR- 18bpm. There was mild pallor, but no jaundice, rash or joint deformity. There was blackish discoloration of her big toe with partially healed necrotic ulcer on top of it, while the toe of left foot showed blackish discoloration but no ulceration. Both were tender to touch. Peripheral pulses were palpable in both upper limbs and lower limbs, although the posterior tibial and dorsalis pedis arteries had low volume. There was no temperature change and sensations were intact. Her systemic examination was entirely unremarkable. DAS-28 was calculated which was 1.8, i.e. remission. Initial labs are shown in Table-I. Doppler ultrasound of legs showed reduced blood flow in left posterior tibial and dorsalis pedis arteries with reduced peak systolic velocities. Due to her persistently increased platelets further work-up was done to evaluate the cause of thrombocytosis. JAK2 V617 mutation analysis was done, which turned out to be positive. Furthermore her bone marrow trephine biopsy was done which showed clusters of mature megakaryocytes with multi-lobulated staghorn nucleoli, suggestive of Essential thrombocytosis. Hence, this patient was diagnosed as a case of RA with associated ET. Treatment was modified in accordance with the revised diagnosis, which included Aspirin 75mg and Hydroxyurea 1g per day, in addition to her RA treatment. Patient responded well to the treatment with regard to clinical as well as laboratory parameters and is on regular follow-up visits, however, she developed gangrene of toe, for which amputation of distal phalanx of toe and nail excision was done, later in the disease course. Anagleride was started with the given treatment due to a surge in her platelet counts. Her follow-up platelet counts are shown in Fig.2.

**Table-I:** Initial laboratory parameters.

	Normal Ranges	Patient’s value
Hb	11-15.5 g/dl	11.6
MCV	80-97 fL	89
Hct	35-50%	28
WBC	4-10*10^9^/L	12.3*10^9^
Platelet	150-450*10^9^/L	1242*10^9^
Peripheral Film	Anisocytosis, microcytosis, thrombocytosis	
CRP	0-5 mg/dl	0.12
Anti-CCP	<5 U/ml	>200
Cr	0.5-1.5 mg/dl	0.58
ALT	8-45 U/L	8
AST	8-33 U/L	12

## DISCUSSION

An increased risk of hematological malignancies has been reported in patients with autoimmune rheumatic diseases, namely RA and SLE.^3^,^4^ Most studies have reported lymphoproliferative disorders in autoimmune rheumatic disease, however myeloid malignancies have also been reported.^5^ Thrombocytosis is frequently seen in Rheumatoid arthritis (RA) in relation to active joint disease. However, persistently elevated platelet counts warrant consideration for further work-up. It has been reported that platelet counts >100000/L should be investigated for colonal malignancies. This patient’s symptoms were initially considered as part of RA associated vasculitis; however, her symptoms of digital ischemia were more towards erythromelalgia rather true Raynaud’s phenomenon. Moreover, no explainable co-relation was evident for high platelets in this patient as her disease activity of RA reflected that she was in remission. There are several other possible explanations for thrombocytosis in RA; reactive as part of active inflammation or extra-articular manifestation of RA or articular features of underlying myeloproliferative disorder as part of paraneoplastic features. But it is important to remember that even for reactive thrombocytosis, platelets count of approximately >600000/L or beyond with no possible explanation, an active surge for myeloproliferative neoplasm such as ET should be thoroughly done. The higher circulating level may lead to spontaneous aggregation of platelets with increase propensity to both arterial and venous thrombosis and ultimately gangrene or other devastating complications such as stroke, TIA, or coronary artery disease etc. A significantly higher incidence of both arterial and venous thromboembolic complications has been reported in patients with primary thrombocytosis. Raised biomarkers of inflammation and chronic autoimmune and inflammatory disorders have been described in many disease entities.^6^ In spite of this, it is not established yet, how these disorders interact and influence each other for disease initiation. There is only one case of ET with RA, reported so far in the literature to the best of our knowledge.^7^Our patient was initially attributed for RA related complications, later her unexplainable persistently raised platelet counts rectify the active surge for clonal malignancy. Moreover, malignant diseases may trigger generation of auto-antibodies against a wide range of antigens as a result of immune dys-regulation. Few studies have reported presence of autoantibodies related to rheumatic diseases, such as anti-Ro, anti-La, anti-Ds DNA, ANA and RF in sera of patient with malignancies.^8^ Whether or not the mere presence of antibodies related to rheumatic diseases could lead to the initiation of an autoimmune disease process in these patients, is not certain as yet.

However, on the other hand there is evidence that persistent inflammation promotes genetic instability. An intriguing possibility is shared genetic polymorphism that predisposes an individual to certain malignancies.^9^-^10^ This case points toward a possible interaction at molecular level, which could act as a trigger for a mutagenic process and it provides an insight to look for any casual factors that might act synergistically. Nonetheless, malignancy related inflammation or vice versa represents a new and challenging target for innovative diagnostic and therapeutic strategies.

## CONCLUSION

This case represents a very rare association between an autoimmune inflammatory rheumatic disease and clonal disorder. It suggests that it is prudent to evaluate every patient with unexplainable thrombocytosis to sought reactive versus clonal hematological malignancies and to prevent devastating complications.

### Author’s Contribution

**WA,** **UE,** **AJM:** Conception and design, drafting, revision and final approval of the manuscript.
